# Homotypic targeting of immunomodulatory nanoparticles for enhanced peripheral and central immunity

**DOI:** 10.1111/cpr.13192

**Published:** 2022-01-27

**Authors:** Yubo Shen, Daoxia Guo, Xiaoyuan Ji, Yanfeng Zhou, Shuo Liu, Jing Huang, Haiyun Song

**Affiliations:** ^1^ 56694 State Key Laboratory of Oncogenes and Related Genes Center for Single‐Cell Omics School of Public Health Shanghai Jiao Tong University School of Medicine Shanghai China; ^2^ Xinyang Normal University Xinyang China; ^3^ Department of Neurology Xuhui District Central Hospital Shanghai China

## Abstract

**Objectives:**

Synthetic oligodeoxynucleotides (ODNs) that contain unmethylated cytosine–phosphate–guanine (CpG) motifs serve as immune adjuvants in disease treatment. However, the poor cell permeability and safety concerns limit their medical applications, and biocompatible strategies for efficient delivery of functional CpG ODNs are highly desirable.

**Materials and Methods:**

Self‐assembled, cell membrane‐coated CpG nanoparticles (NP) are prepared, and their physicochemical properties are characterized. The uncoated and membrane‐coated CpG NP are compared for their biocompatibility, cellular uptake kinetics, endocytic pathways, subcellular localization, and immunostimulatory activities in macrophages and microglia.

**Results:**

Macrophage‐ or microglia‐derived cell membrane camouflaging alters the endocytic pathways of CpG NP, promotes their targeted delivery to the cells with homologous membrane, ensures their endosomal localization, and enhances their immunomodulatory effects.

**Conclusions:**

We design a type of biomimetic NP consisting of self‐assembled CpG NP core and cell membrane shell, and demonstrate its advantages in the modulation of peripheral and central immune cells. Our study provides a new strategy for the application of CpG ODNs.

## Graphical abstract

Cell membrane camouflage switches the endocytic pathways of self‐assembled CpG nanoparticles, promotes their delivery to macrophages or microglia via homotypic targeting, and enhances their immunostimulatory effects on cytokine production for the modulation of peripheral or central immune cells.

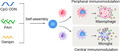

## 1 INTRODUCTION

Unmethylated cytosine–phosphate–guanine (CpG) dinucleotide motifs belong to a classic type of pathogen‐associated molecular patterns (PAMPs) and exist extensively in the genomic DNA of bacteria and viruses.^1^, ^2^, ^3^, ^4^ The recognition of exogenous CpG motifs is mediated by the Toll‐like receptor 9 (TLR9) of host cells.^3^, ^5^, ^6^ As a class of host innate immune response, subsequent TLR9 activation primes a series of signal transduction, at least in part via the nuclear factor κ‐light‐chain enhancer of activated B cells (NF‐κB) pathway and the mitogen‐activated protein kinase (MAPK) pathway, inducing the expression of proinflammatory cytokines such as tumor necrosis factor‐α (TNF‐α) and interleukin‐6 (IL‐6).^7^, ^8^, ^9^ Synthetic oligodeoxynucleotides (ODNs) comprised CpG motifs are found to stimulate similar immunomodulatory responses and display a great potential in the therapy of infection, cancer, or allergy.^5^, ^10^, ^11^ However, free CpG ODNs are susceptible to nuclease cleavage and have poor cell permeability due to their anionic and hydrophilic properties.^12^, ^13^, ^14^, ^15^, ^16^ Although phosphorothioate modification of the backbone can confer the resistance to nuclease degradation, it concurrently increases the cytotoxicity and thereby limits the clinical utilization of CpG ODNs.^17^, ^18^, ^19^, ^20^ Therefore, it is highly appealing to develop efficient and safe delivering strategies for CpG ODNs, improving their bioavailability and biocompatibility.

Nanoparticles (NP) have been widely explored as carriers of small molecule chemicals and biomolecules.^21^, ^22^, ^23^, ^24^, ^25^, ^26^ Recent studies have demonstrated their superior advantages in the delivery of functional nucleic acids including short interfering RNA (siRNA), viral RNA mimics, and CpG ODNs. As an example, a tumor microenvironment‐responsive nanobooster encapsulates a hybrid RNA encoding both siRNA and viral RNA signature for orchestrated activation of innate immunity and potent induction of long‐term T cell memory, eliciting multilayer antitumor activities.^27^ Many types of nanodevices have been designed for the loading of CpG ODNs. Among them, Gold NP represent one kind of convenient option as they can form stable interaction with CpG ODNs containing either thiolated terminal or polyadenine sequence.^19^, ^28^ Besides, CpG ODNs can be easily tethered to DNA nanostructures via complementary base pairing.^29^ Despite such progress, nanocarriers that aim for more efficient packaging and precise delivery of CpG ODNs are still in great demand.

Although nanocarriers are often modified with targeting molecules as a guidance to specific organs or tissues, the formation of protein corona on the surface of NP can compromise the delivery efficiency.^30^, ^31^, ^32^, ^33^ Recently, camouflage with natural cell membrane has become an advanced strategy for surface functionalization of NP.^34^, ^35^, ^36^, ^37^, ^38^, ^39^ Cell membrane‐coated NP possess many unique properties of the source cells, especially the capacity of homotypic targeting that allows for active accumulation of NP in their desired destination. In addition, surface coating with cell membranes can greatly enhance the biocompatibility and circulation lifetime of NP and promote their cellular uptake.^40^, ^41^, ^42^

**FIGURE 1:**
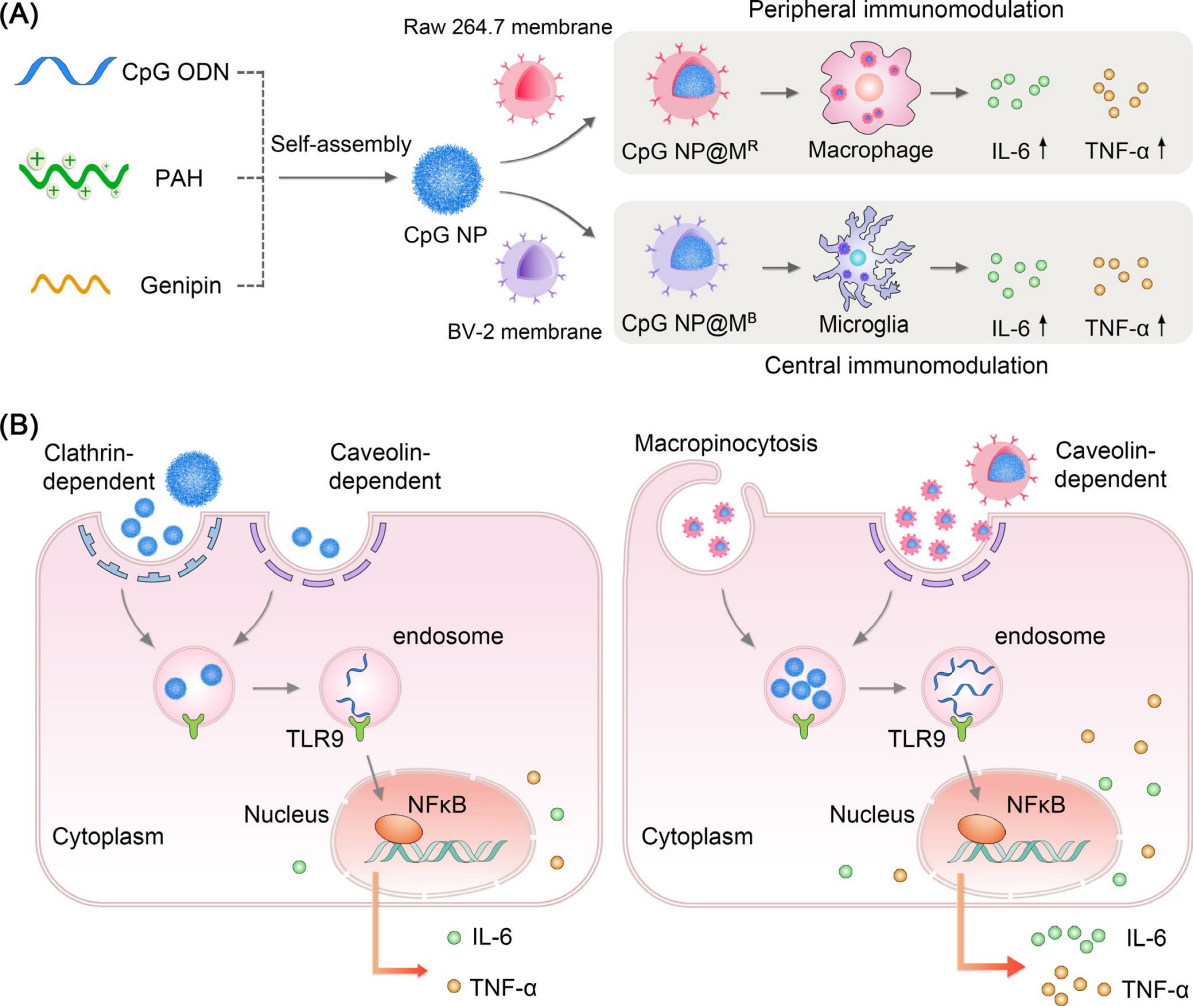
Schematic illustration of homotypically targeted nanoparticles for peripheral and central immunomodulation. (A) Design and assembly of CpG NP, camouflaged with the membranes from Raw264.7 macrophages or BV‐2 microglia (CpG NP@M^R^ or CpG NP@M^B^), for the regulation of peripheral or central immune systems. (B) Schematic diagrams for the endocytic pathways of CpG NP and CpG NP@M

Herein, we utilize one‐step self‐assembly of CpG NP, which are further camouflaged with the cell membrane of macrophages or microglia, for targeted immunomodulation in peripheral or central immune system (Figure 1A). Compared to uncoated CpG NP, the Raw264.7 macrophage cell membrane‐coated NP (CpG NP@M^R^) and the BV‐2 microglial cell membrane‐coated NP (CpG NP@M^B^) display significantly increased levels of cellular internalization. In addition, the cell membrane coating switches the endocytosis of CpG NP from a clathrin‐dependent pathway to caveolae‐ and macropinocytosis‐dependent pathways. Nevertheless, the majority of cell membrane‐coated CpG NP are delivered to the endolysosomal compartments, allowing for TLR9 recognition and subsequent signaling activation. Accordingly, both CpG NP@M^R^ and CpG NP@M^B^ stimulate higher expression levels of proinflammatory cytokines (TNF‐α and IL‐6) than uncoated CpG NP in macrophages and microglia, respectively (Figure 1B).

## 2 MATERIALS AND METHODS

### 2.1 Cell culture and reagents

Raw264.7 macrophages were purchased from the Cell Bank of Chinese Academy of Sciences (Shanghai). BV‐2 microglia were obtained from Fuheng Biology Science and Technology Co., Ltd. The Raw264.7 cells and BV‐2 cells were cultured with the Dulbecco's modified Eagle medium (DMEM, from Gibco) in a humidified incubator at 37 °C and with 5% CO_2_. The media were supplemented with 10% fetal bovine serum (FBS, from Gibco), 100‐U/ml streptomycin (Invitrogen), and 100‐U/ml penicillin (Invitrogen). CpG ODN (5′‐TCCATGACGTTCCTGACGTT‐3′) was from BioSune Biotechnology Co. Ltd. Poly (allylamine hydrochloride) (PAH) (15 kDa), genipin, chlorpromazine hydrochloride (CPZ), methyl‐β‐cyclodextrin (MβCD), and 5‐(N‐methyl‐N‐isobutyl) amiloride (MIBA) were from Sigma‐Aldrich. LysoTracker™ Deep Red was from Invitrogen. 4’,6‐Diamidino‐2‐phenylindole dihydrochloride (DAPI) and Cell Counting Kit‐8 (CCK‐8) were from Beyotime. The TNF‐α and IL‐6 enzyme‐linked immunosorbent assay (ELISA) kits were from Jianglai Industrial Co., Ltd.

### 2.2 Preparation and characterization of CpG NP

CpG ODNs and PAH were utilized to assemble CpG NP via crosslinking with genipin. In brief, 2‐mg CpG ODNs and 9‐mg genipin were dissolved in 2.8‐ml Milli‑Q water and stirred for 24 h. The mixture was diluted to 14 ml, slowly dripped into 10‐ml PAH solution (Mw = 15 kDa, 0.3 mg/ml), and further stirred for 24 h. The assembled CpG NP were purified by centrifugation and washing with Milli‑Q water. Agarose gel electrophoresis was utilized to confirm the formation and stability of CpG NP. The hydrodynamic diameter and zeta potential analysis were carried out on a ZEN3690 Zetasizer (Malvern).

### 2.3 Preparation and characterization of CpG NP@M

Raw264.7 or BV‐2 cells were collected and washed three times with phosphate buffer saline (PBS). To obtain membrane‐derived vesicles, the cells were dispersed and ground in a low permeability buffer containing protease inhibitor cocktails. The supernatant was collected after centrifugation and extruded 15 times through a 400‐nm polycarbonate porous membrane with the assistance of an Avanti mini‐extruder. For cell membrane coating of the CpG NP, the Raw264.7‐ or BV‐2‐derived vesicles (12 *μ*l) were mixed with the CpG NP (CpG ODNs: 12 *μ*g) in 240‐*μ*l Milli‑Q water, sonicated for 5 min, and incubated at 4°C overnight. The obtained CpG NP@M were characterized with dynamic light scattering and zeta potential analysis.

### 2.4 Cell viability assays

Cells were dispersed in 96‐well plates at a density of 5 × 10^3^ cells per well and cultured overnight at 37°C. After incubation with CpG NP or CpG NP@M (CpG ODNs: 0.25–2 *μ*g/ml) for 48 h, the cells were treated with fresh media containing 10% CCK‐8 solution for 30 min at 37°C. The viabilities of the cells were determined by detecting the absorbance around 450 nm with a microplate reader.

### 2.5 Cellular internalization of CpG NP and CpG NP@M

To study the cellular uptake of CpG NP and CpG NP@M, the 6‐carboxyfluorescein (FAM)‐labeled CpG ODNs were employed to prepare fluorescent CpG^FAM^ NP and CpG^FAM^ NP@M. Raw264.7 cells or BV‐2 were dispersed in 24‐well plates overnight at a density of 1.0 × 10^5^ cells per well, followed by incubation with the CpG NP or CpG NP@M (CpG ODNs: 1 *μ*g/ml) for various periods (1, 3 and 6 h). The cells were fixed with 4% paraformaldehyde (PFA), washed with PBS, and stained with DAPI (2.5 *μ*g/ml) to label the nuclei. The cellular internalization of CpG^FAM^ NP and CpG^FAM^ NP@M was monitored by confocal laser scanning microscopy (CLSM, Leica TCS SP8) imaging and quantified with the Leica Application Suite Advanced Florescence Lite (LAS AF Lite) software. All the images were captured under the same instrument settings.

### 2.6 Endocytic pathway analysis

Raw264.7 cells were preincubated with one of the endocytosis inhibitors: CPZ (10 *μ*g/ml), MβCD (2.5 mg/ml), or MIBA (12.5 *μ*g/ml) for 30 min. Thereafter, CpG^FAM^ NP or CpG^FAM^ NP@M^R^ were added into the cell culture and further incubated for 6 h. The cells were fixed with 4% PFA, washed with PBS, and stained with DAPI (2.5 *μ*g/ml). CLSM imaging and fluorescence quantification were performed to determine the levels of cellular uptake for CpG^FAM^ NP and CpG^FAM^ NP@M^R^ under the influence of different inhibitors.

### 2.7 Colocalization assays

Raw264.7 cells were dispersed in 24‐well plates for 12 h at a density of 1.0 × 10^5^ cells per well, followed by incubation with fluorescent CpG^FAM^ NP or CpG^FAM^ NP@M^R^ (CpG ODNs: 1 *μ*g/ml) for 6 h. The cells were incubated with 250‐nM LysoTracker™ Deep Red for 40 min. Thereafter, the cells were fixed with 4% PFA, washed twice with PBS, and stained with DAPI (2.5 *μ*g/ml). CLSM imaging was utilized to monitor the intracellular localization of fluorescent CpG^FAM^ NP or CpG^FAM^ NP@M^R^. The ratios of colocalization with the endolysosomes were calculated with the Image J software.

### 2.8 Cytokine assays

Raw264.7 or BV‐2 cells were dispersed at a density of 0.5 × 10^5^ cells per well in 24‐well plates and treated with isolated cell membrane‐derived vesicles, CpG ODNs, CpG NP, or CpG NP@M (CpG ODNs: 2 *µ*g/ml). After incubation for 48 h, the supernatants were collected and centrifuged at 13,523 *g* for 10 min. The purified supernatants were used for ELISA assays following the protocols provided by the manufacturers.

### 2.9 Statistical analysis

The results were presented as mean ± standard deviation (s.d.) or standard error of the mean (s.e.m.) from three or more independent samples. The Student's *t*‐test was used to determine the statistical significance of the differences between two groups.

## 3 RESULTS

### 3.1 Preparation and characterization of cell membrane‐coated CpG NP

CpG ODNs and PAH were utilized to fabricate the self‐assembled CpG NP via crosslinking with genipin. The stability of self‐assembled CpG NP was tested in a gel retardation electrophoresis. Free CpG ODNs migrated in the agarose gel in a time‐dependent manner, whereas the CpG NP were retained in the well without the detection of dissembled CpG ODNs (Figure S1). The hydrodynamic diameter of CpG NP was ~140 nm, with a positive surface charge (33.9 ± 4.7 mV). The Raw264.7 cell membrane‐camouflaged CpG NP@M^R^ displayed a slightly increased size (~160 nm) and negatively charged surface (−9.5 ± 0.5 mV) (Figure S2A,B). These changes in physicochemical properties confirmed successful coating of the CpG NP by the cell membrane. We also monitored the stability of CpG NP and CpG NP@M^R^ under different physiological contexts. CpG NP showed a little increase of the hydrodynamic diameter in PBS (~165 nm) and the DMEM cell culture medium (~190 nm) (Figure S2C). In contrast, these solutions had very subtle influence on the size of CpG NP@M^R^ (160 nm in water vs. 174 nm in PBS and 169 nm in DMEM), suggesting a more stable conformation for the membrane‐coated form of CpG NP (Figure S2D).

### 3.2 Cellular uptake efficiency

**FIGURE 2:**
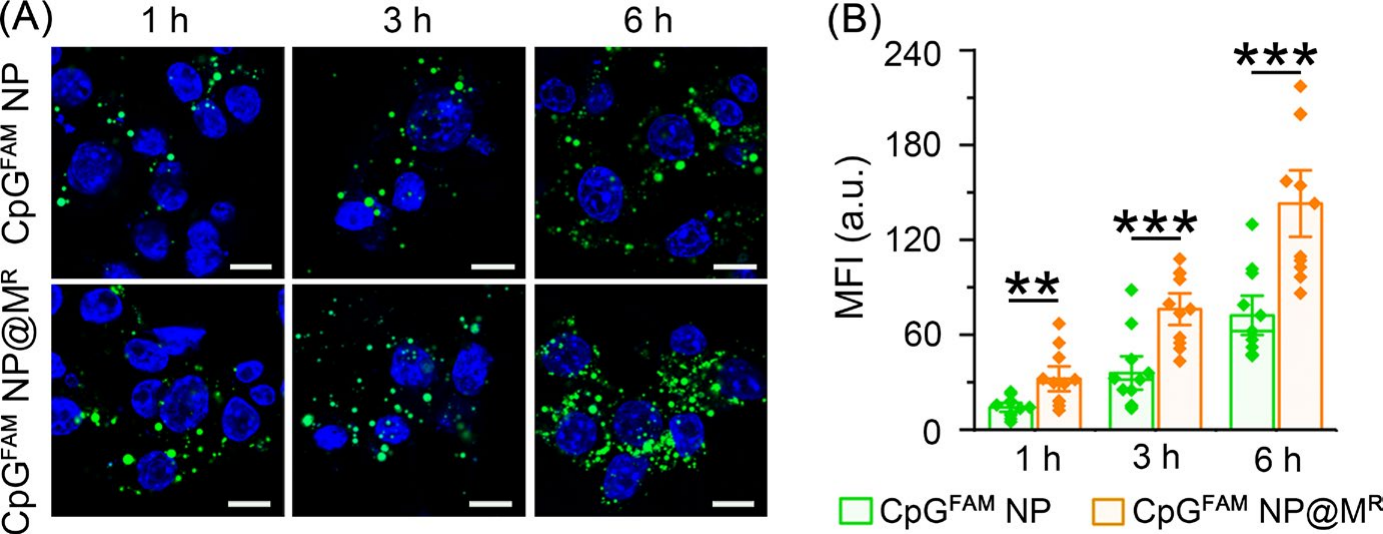
Time‐dependent uptake of CpG^FAM^ NP and CpG^FAM^ NP@M^R^ by Raw264.7 cells. (A) Confocal images of internalized CpG^FAM^ NP and CpG^FAM^ NP@M^R^ (CpG^FAM^: 1 *μ*g/ml) at different time points. Scale bars, 10 *μ*m. (B) The geometric mean fluorescence intensities (MFI) of internalized nanoparticles assessed with the Leica LAS AF Lite software. Data are represented as mean ± s.e.m. (*n =* 10). Student's *t*‐test, **p < 0.01, ***p < 0.001

Coating with cell membrane can confer NP with homotypic affinity that is mediated by adhesive interactions between specific cell surface proteins, enhancing the binding of camouflaged NP to the same type of cells.^43^, ^44^ We thus speculated that the coating of macrophage membrane would promote the cellular uptake of CpG NP by Raw264.7 cells. First, we assessed the biocompatibility of CpG NP and CpG NP@M^R^ in Raw264.7 cells. Both types of NP exhibited negligible cytotoxicity at the concentrations ranging from 0.25–2 *μ*g/ml after 48‐hour incubation (Figure S3). Next, the kinetics of cellular internalization was investigated using the types of NP consisting of fluorescent dye‐conjugated CpG ODNs (CpG^FAM^ NP and CpG^FAM^ NP@M^R^). A time‐dependent internalization of CpG^FAM^ NP was observed in the Raw264.7 cells in a time window of 24 h, with obviously accelerated cellular uptake occurring between the 12th and the 24th hour (Figure S4). The CpG^FAM^ NP@M^R^ demonstrated a significantly faster speed of internalization than the CpG^FAM^ NP after various periods of incubation (1, 3 and 6 h). At each time point after incubation, the levels of fluorescence signal from the internalized CpG^FAM^ NP@M^R^ were at least one‐fold higher than that of the CpG^FAM^ NP (Figure 2). These results confirmed that the macrophage membrane‐camouflaged CpG NP could achieve faster and higher levels of cellular internalization than their uncoated counterparts via homotypic targeting.

### 3.3 Endocytic pathways

**FIGURE 3:**
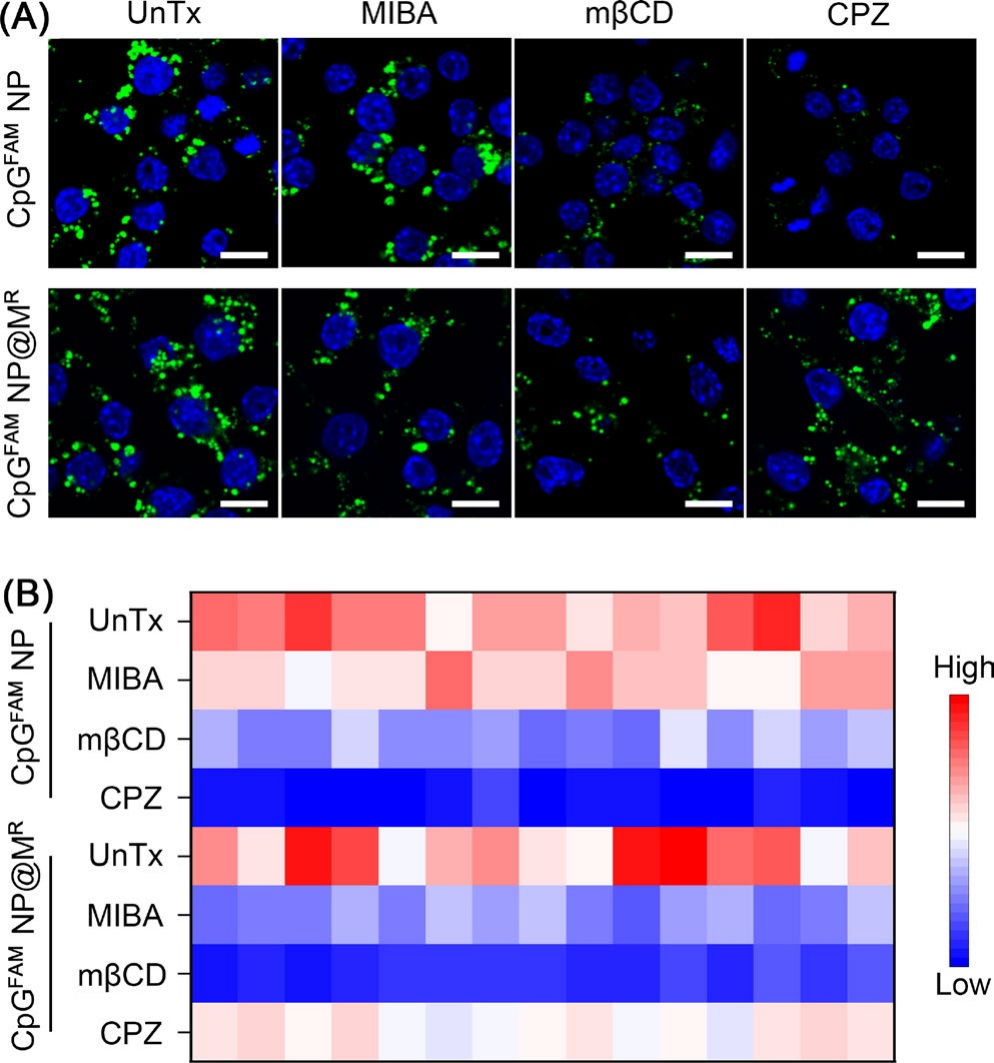
Endocytic pathways for internalization of CpG NP and CpG NP@M in Raw264.7 cells. (A) Confocal images of CpG^FAM^ NP and CpG^FAM^ NP@M^R^ (CpG^FAM^: 1 *μ*g/ml) in Raw264.7 cells pretreated with MIBA, MβCD, or CPZ. Scale bars, 10 *μ*m. (B) Color‐coded grid showing normalized MFI of internalized nanoparticles assessed with the Leica LAS AF Lite software. Colors ranging from blue to red represent the lowest and the highest intensity, respectively

The different internalization kinetics of CpG^FAM^ NP and CpG^FAM^ NP@M^R^ triggered our interest to explore whether the membrane coating affected their endocytic pathways in the Raw264.7 cells. We preincubated the cells with well‐established pharmacological inhibitors of endocytosis, including MIBA for the inhibition of macropinocytosis, MβCD for the inhibition of caveolae‐mediated endocytosis, and CPZ for the inhibition of clathrin‐mediated endocytosis. Treatment with MIBA had a very mild influence on cellular internalization of CpG^FAM^ NP. Instead, MβCD moderately reduced the signal of CpG^FAM^ NP by 47.7% ± 2.5%. Moreover, the levels of internalization in the CPZ‐treated group were reduced by 87.8% ± 1.3%, indicating that the clathrin‐mediated endocytosis played a predominant role in cellular uptake of CpG^FAM^ NP in macrophages (Figure 3 and Figure S5A). In comparison, CPZ only had a minor effect on the internalization of CpG^FAM^ NP@M^R^. Macropinocytosis serves as a primary method for cellular uptake of membrane‐bound cargoes.^45^ Consistently, the contribution of macropinocytosis was largely increased in the uptake of CpG^FAM^ NP@M^R^, and MIBA treatment reduced the levels of fluorescence signal by 54.7% ± 2.3%. In addition, MβCD treatment reduced the signal of CpG^FAM^ NP@M^R^ by 78.9% ± 1.4% (Figure 3 and Figure S5B). These results suggested that the camouflage with cell membrane shifted the endocytosis of CpG NP from a clathrin‐mediated pathway to caveolae‐ and micropinocytosis‐mediated pathways.

### 3.4 Intracellular trafficking

**FIGURE 4:**
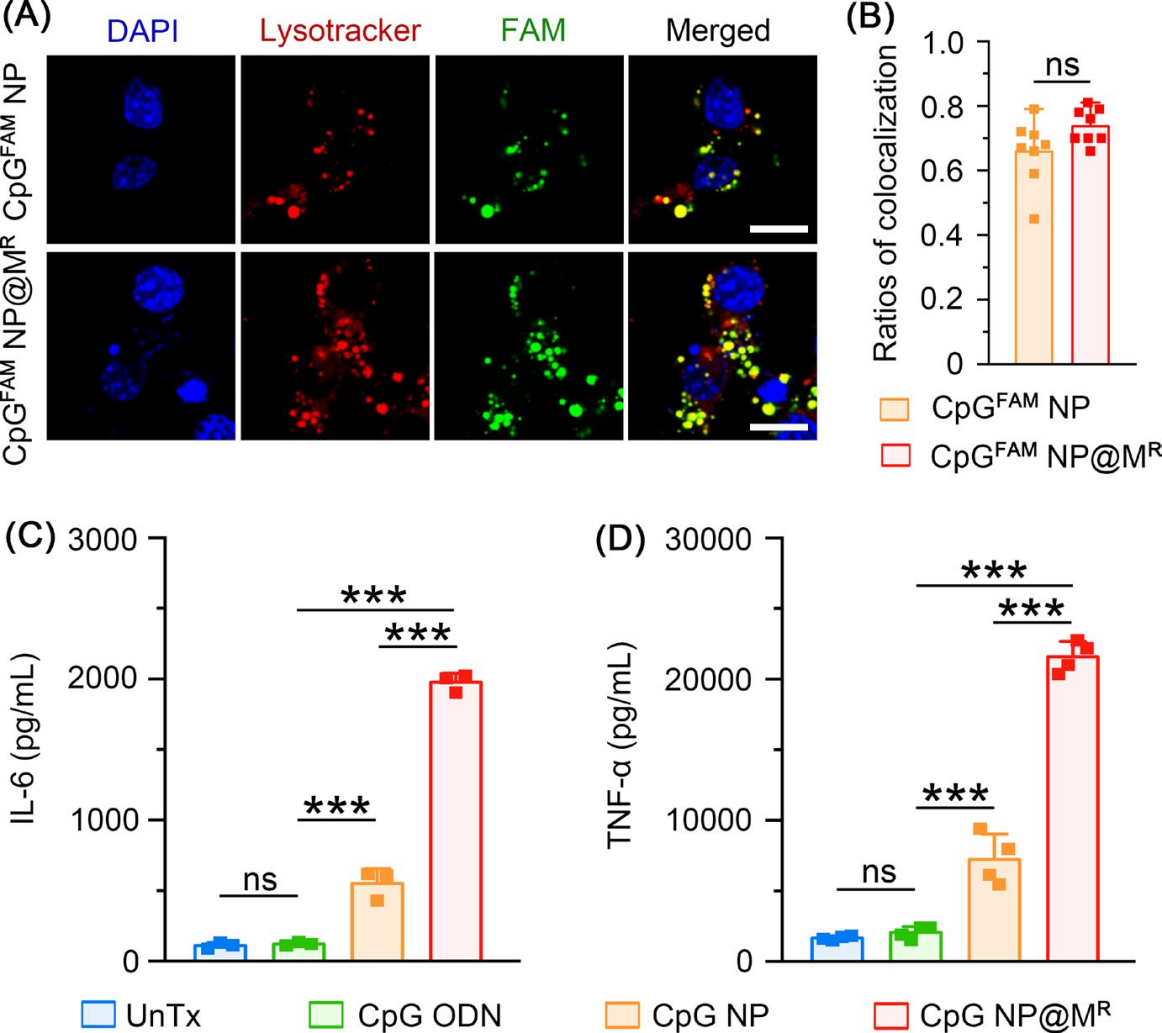
Immune activation of macrophages via homotypically targeted nanoparticles. (A) Live imaging of intracellular CpG^FAM^ NP and CpG^FAM^ NP@M^R^ (CpG^FAM^: 1 *μ*g/ml) in Raw264.7 cells after 6‐hour incubation. Scale bars, 10 *μ*m. (B) The ratios of CpG^FAM^ NP and CpG NP@M^R^ colocalized with the lysosomes. Data are represented as mean ± s.e.m. (*n =* 8). Student's *t*‐test, ns means not significant. (C and D) Secreted IL‐6 (C) and TNF‐α (D) from Raw264.7 cells treated with CpG ODN, CpG NP, or CpG NP@M^R^ (CpG: 2 *μ*g/ml) for 48 h. Data are represented as mean ± s.d. (*n* ≥ 3). Student's *t*‐test, ns means not significant, ***p < 0.001

The TLR9 receptors are localized on the membrane of endosomal compartments, where they interact with internalized CpG ODNs for signaling activation.^46^, ^47^, ^48^, ^49^ We therefore examined the subcellular localization of CpG^FAM^ NP and CpG^FAM^ NP@M^R^. LysoTracker is a fluorescent dye that labels acidic organelles such as the endolysosomes. We monitored the ratios of CpG^FAM^ NP and CpG^FAM^ NP@M^R^ that were colocalized with the LysoTracker signal. Although the uncoated and cell membrane‐coated NP adopted different endocytic pathways, they both showed high and similar levels of endosomal localization (0.66 ± 0.04 vs. 0.74 ± 0.02), ensuring their interaction with TLR9 (Figure 4A,B).

### 3.5 Peripheral and central immunomodulation

Next, we examined the immunomodulatory activities of uncoated CpG NP and membrane‐coated CpG NP@M^R^ in macrophages. The RAW264.7 cells were incubated with free CpG ODNs, CpG NP, or CpG NP@M^R^ that contained equal amounts of nucleic acids (2 *μ*g/ml) and were measured for the production of proinflammatory cytokines including IL‐6 and TNF‐α. Free CpG ODNs displayed negligible immunostimulatory effects on the secretion of IL‐6 or TNF‐α due to their poor cell permeability. In contrast, both CpG NP and CpG NP@M^R^ potently induced IL‐6 and TNF‐α secretion. More importantly, the levels of cytokine production in the CpG NP@M^R^ group were several fold higher than that in the CpG NP group, validating the high efficiency of CpG NP@M^R^ for peripheral immunomodulation (Figure 4C,D). The enhanced immunostimulatory effects might attribute to the faster cellular uptake efficiency of CpG NP@M^R^, as the equivalent amount of NP‐bound cell membrane was unable to provoke the production of IL‐6 or TNF‐α by itself (Figure S6).

**FIGURE 5:**
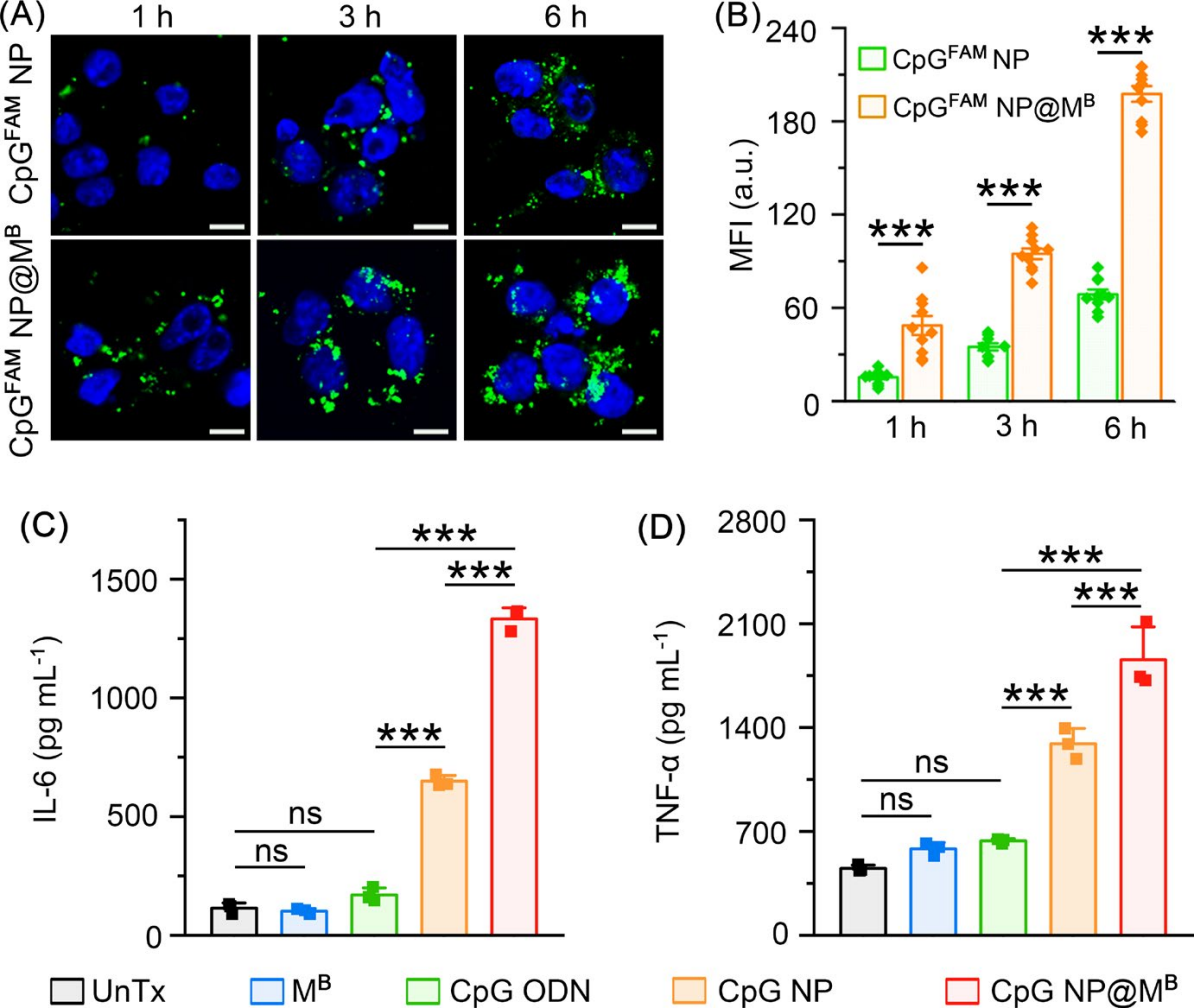
Immune activation of microglia via homotypically targeted nanoparticles. (A) Confical images of internalized CpG^FAM^ NP and CpG^FAM^ NP@M^B^ (CpG^FAM^: 1 *μ*g/ml) in BV‐2 microglia at different time points. Scale bars, 10 *μ*m. (B) MFI of internalized nanoparticles assessed with the Leica LAS AF Lite software. Data are represented as mean ± s.e.m. (*n =* 10). Student's *t*‐test, ***p < 0.001. (C,D) Secreted IL‐6 (C) and TNF‐α (D) from BV‐2 cells incubated with the BV‐2 cell membrane (M^B^), CpG ODN, CpG NP, or CpG NP@M^B^ (CpG: 2 *μ*g/ml) for 48 h. Data are represented as mean ± s.d. (*n =* 3). Student's *t*‐test, ns means not significant, ***p < 0.001

Microglia are tissue‐resident macrophages in the central nervous system and are activated during brain infection, injury, or inflammation.^50^, ^51^, ^52^, ^53^ We further investigated the capacities of uncoated CpG NP and BV‐2 microglial cell membrane‐coated CpG NP (CpG NP@M^B^) to modulate immunity in the microglia. After camouflaging with the BV‐2 cell membrane, the average hydrodynamic diameter of CpG NP was increased from ~140 nm to ~160 nm, accompanied by a reversion of surface charge from 34.0 ± 1.0 mV to −6.9 ± 1.0 mV (Figure S7). We evaluated the biocompatibility of CpG NP and CpG NP@M^B^ in BV‐2 cells. Neither type of NP demonstrated noticeable cytotoxicity at the concentrations ranging from 0.25–2 *μ*g/ml after 48‐hour incubation (Figure S8). Using fluorescent dye‐conjugated CpG^FAM^ NP and CpG^FAM^ NP@M^B^, we compared the cellular uptake of these two forms of NP by BV‐2 microglia. CpG^FAM^ NP@M^B^ were internalized much faster than CpG^FAM^ NP at various time points after incubation, validating the effectiveness of cell membrane coating in the enhancement of internalization efficiency (Figure 5A,B). Finally, we monitored the production of proinflammatory cytokines in the microglia stimulated with the BV‐2 cell membrane (M^B^), free CpG ODNs, CpG NP, or CpG NP@M^B^. Neither M^B^ nor free CpG ODNs stimulated the secretion of IL‐6 or TNF‐α, whereas CpG NP and CpG NP@M^B^ significantly enhanced it. Notably, CpG NP@M^B^ induced the cytokine secretion more efficiently than CpG NP, implying its superiority in the regulation of central immunity (Figure 5C,D).

## 4 DISCUSSION

The potential applications of CpG ODNs are limited due to their susceptibility to biodegradation and poor efficiency in cell permeability. Herein, a type of one‐step self‐assembled CpG NP was designed. The CpG NP were further coated with the Raw264.7 macrophage cell membrane‐ or the BV‐2 microglia cell membrane‐derived vesicles for homotypic targeting to these two classes of immune cells. Either type of membrane coating affected the physicochemical properties of CpG NP in a similar way and resulted in good biocompatibility. Besides, membrane coating led to accelerated rates in the cellular internalization of CpG NP@M^R^ and CpG NP@M^B^. By our observation, the levels of internalized membrane‐coated NP were at least one‐fold higher than that of uncoated NP at various time points, confirming the feasibility of homologous membrane camouflage in the improvement of targeted delivery of CpG ODNs. Accompanied with this observation, we found different endocytic pathways for uncoated and membrane‐coated NP. While CpG NP were internalized predominantly via clathrin‐mediated endocytosis, cellular uptake of membrane‐coated CpG NP mainly relied on both caveolae‐ and micropinocytosis‐mediated pathways.

After cellular internalization, the majority (~70%) of both uncoated and membrane‐coated CpG NP trafficked to the endolysosomal compartments, allowing for their interaction with the TLR9 receptors. However, since the levels of internalized CpG NP were much lower than that of their membrane‐coated counterparts, we observed higher levels of CpG NP@M^R^ localized in the endolysosomes. Consistently with this finding, the membrane‐camouflaged CpG NP induced higher levels of IL‐6 and TNF‐α in macrophages and microglia, confirming their advantages in provoking immunostimulatory effects.

In conclusion, we designed a type of biomimetic NP, composed of self‐assembled CpG NP core and cell membrane shell derived from macrophages or microglia, for the modulation of peripheral and central immunity. The utilization of cell membrane camouflage facilitated the targeted delivery of CpG NP to the cells with homologous membrane components, leading to more effective immune activation in peripheral and central immune cells. Our study thus provides a strategy for the application of CpG ODNs as a type of efficient and biocompatible immune adjuvant in disease treatment.

## CONFLICT OF INTEREST

All authors of this paper declare no conflict of interest.

## AUTHOR CONTRIBUTIONS

H.S. and J.H. designed and supervised the research; Y.S. and D.G. performed the experiments; H.S. and D.G. wrote the manuscript; X.J., Y.Z., and S.L. assisted the experiments on nanoparticle synthesis, cytokine assays, and data analysis.

## Supporting information

Figures S1‐S8

## Data Availability

The data supporting the findings of this study are available from the corresponding authors upon reasonable request.
