# Supplementary material for: Homotypic targeting of immunomodulatory nanoparticles for enhanced peripheral and central immunity
Source: Cell Prolif. 2022 Jan 27;55(3):e13192. doi: 10.1111/cpr.13192 (PMC8891550; doi:10.1111/cpr.13192)
Supplement: Supplementary file 1 — Figures S1‐S8 [file CPR-55-e13192-s001.docx]

**Supporting Information**

**Homotypic targeting of immunomodulatory nanoparticles for enhanced** **peripheral and central immunity**

Yubo Shen,^1,‡^ Daoxia Guo,^1,‡^ Xiaoyuan Ji,^1^ Yanfeng Zhou,^1^ Shuo Liu,^2^

Jing Huang,^3,^* Haiyun Song^1,^*

^1^State Key Laboratory of Oncogenes and Related Genes, Center for Single-Cell Omics, School of Public Health, Shanghai Jiao Tong University School of Medicine, Shanghai 200025, China

^2^Xinyang Normal University, Xinyang 464000, China

^3^Department of Neurology, Xuhui District Central Hospital, Shanghai 200032, China

*Correspondence: Haiyun Song, [songhaiyun@shsmu.edu.cn](mailto:songhaiyun@shsmu.edu.cn)

Jing Huang, [huangjing.doc@hotmail.com](mailto:huangjing.doc@hotmail.com)

^‡^These authors contributed equally to this work.


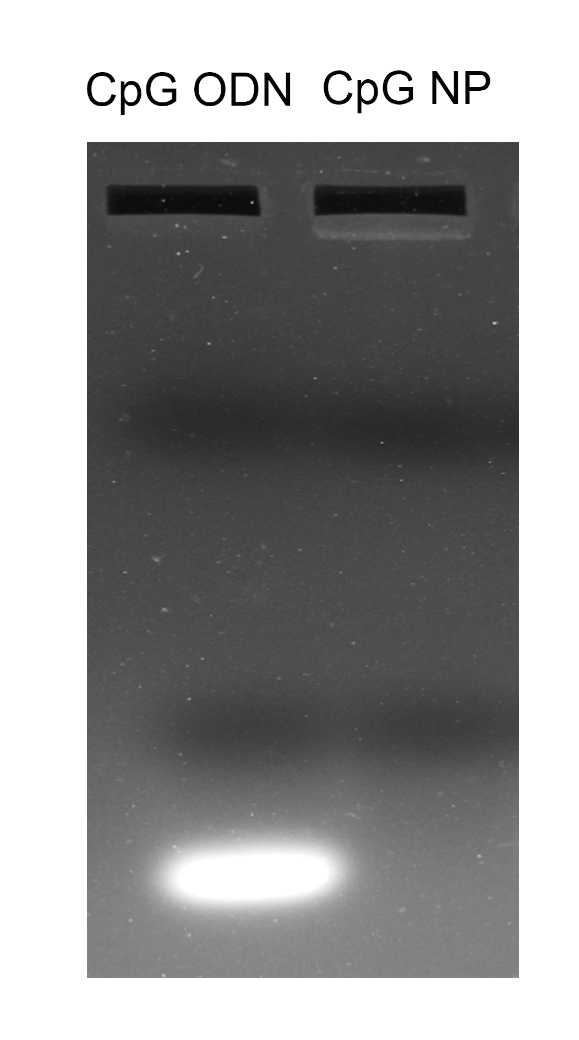


**Figure S1 Characterization of self-assembled CpG NP.** Gel retardation electrophoresis of free CpG ODN and CpG NP (CpG: 200 ng).


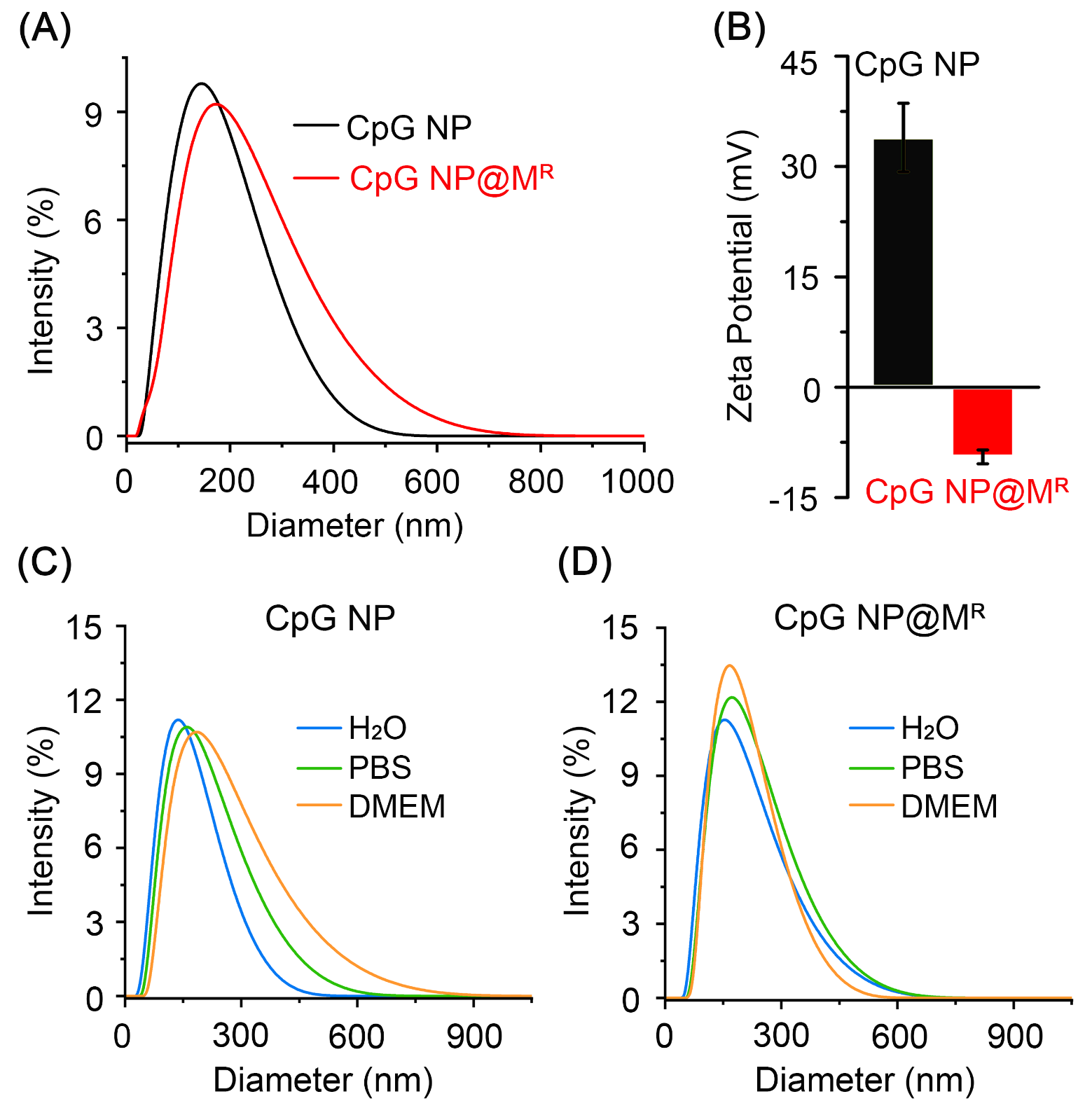


**Figure S2 Characterization of CpG NP and CpG NP@M^R^.** (A) Dynamic light scattering (DLS) analysis for the hydrodynamic diameters of CpG NP and CpG NP@M^R^. (B) Zeta potetials of CpG NP and CpG NP@M^R^. Data are represented as mean ± s.d. (n=3). (C) The hydrodynamic diameters of CpG NP in water, PBS and DMEM. (D) The hydrodynamic diameters of CpG NP@M^R^ in water, PBS and DMEM.


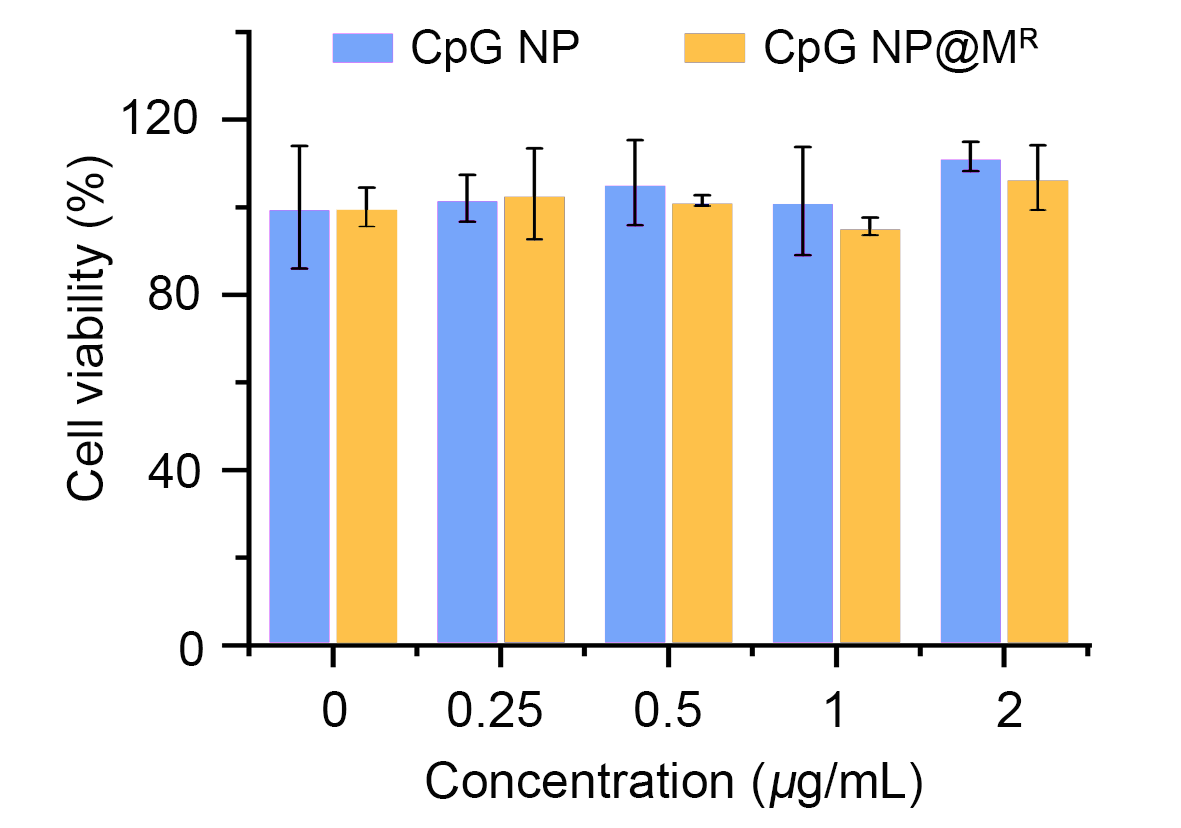


**Figure S3 Biocompatibility of** **CpG NP and CpG NP@M^R^ in Raw264.7 cells.** Cell viabilities of Raw264.7 cells incubated with CpG NP or CpG NP@M^R^ for 48 hours. Data are represented as mean ± s.d. (n=3).


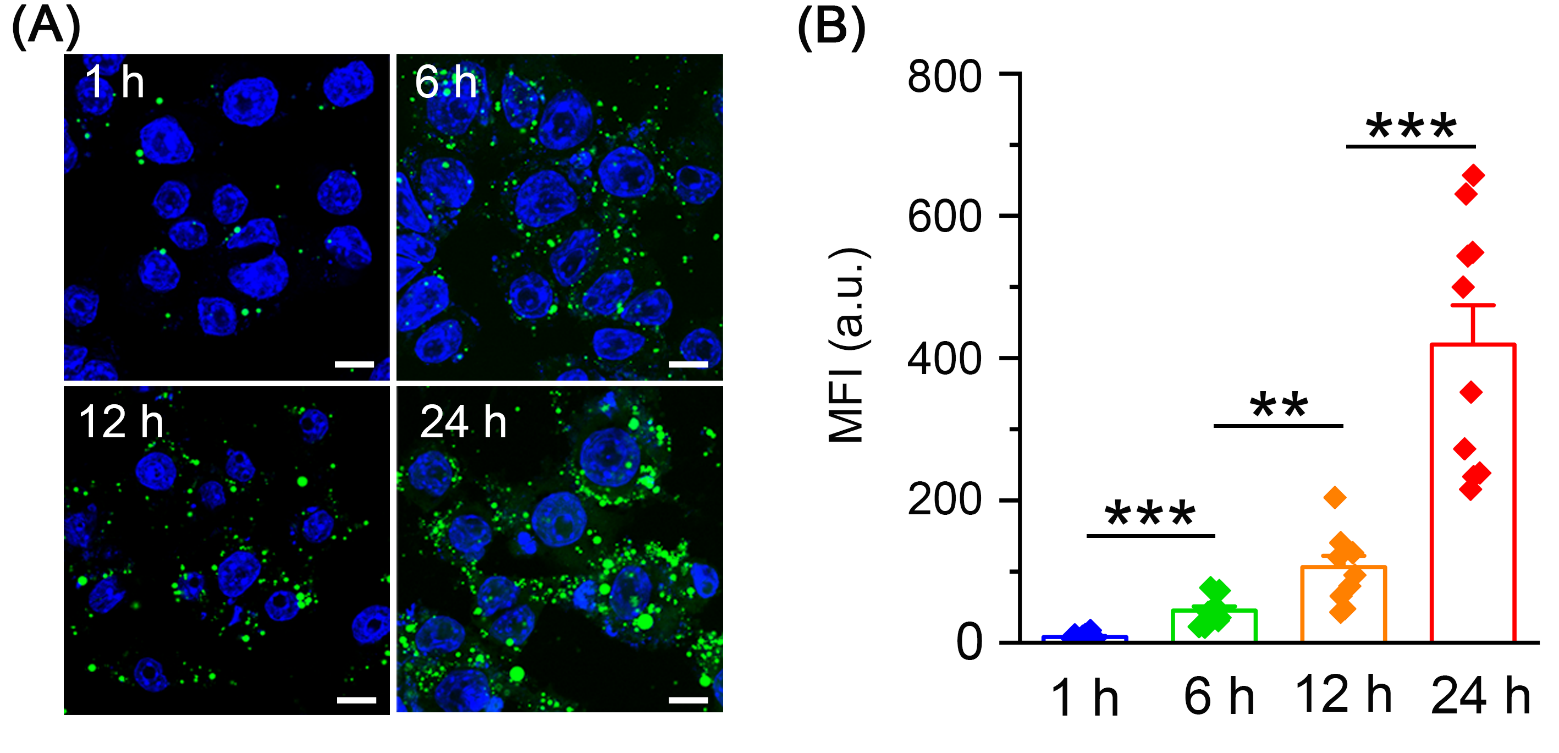


**Figure S4 Time-dependent uptake of CpG NP by Raw264.7 cells.** (A) Confocal images of internalized CpG NP (CpG: 1 *μ*g/mL) at different time points from 1 to 24 h. Scale bars, 10 *μ*m. (B) The geometric mean fluorescence intensities (MFI) of intertnalized CpG NP quantified via the Leica LAS AF Lite software. Data are represented as mean ± s.e.m. (n = 10). Student’s *t*-test, ***p* < 0.01, ****p* < 0.001.


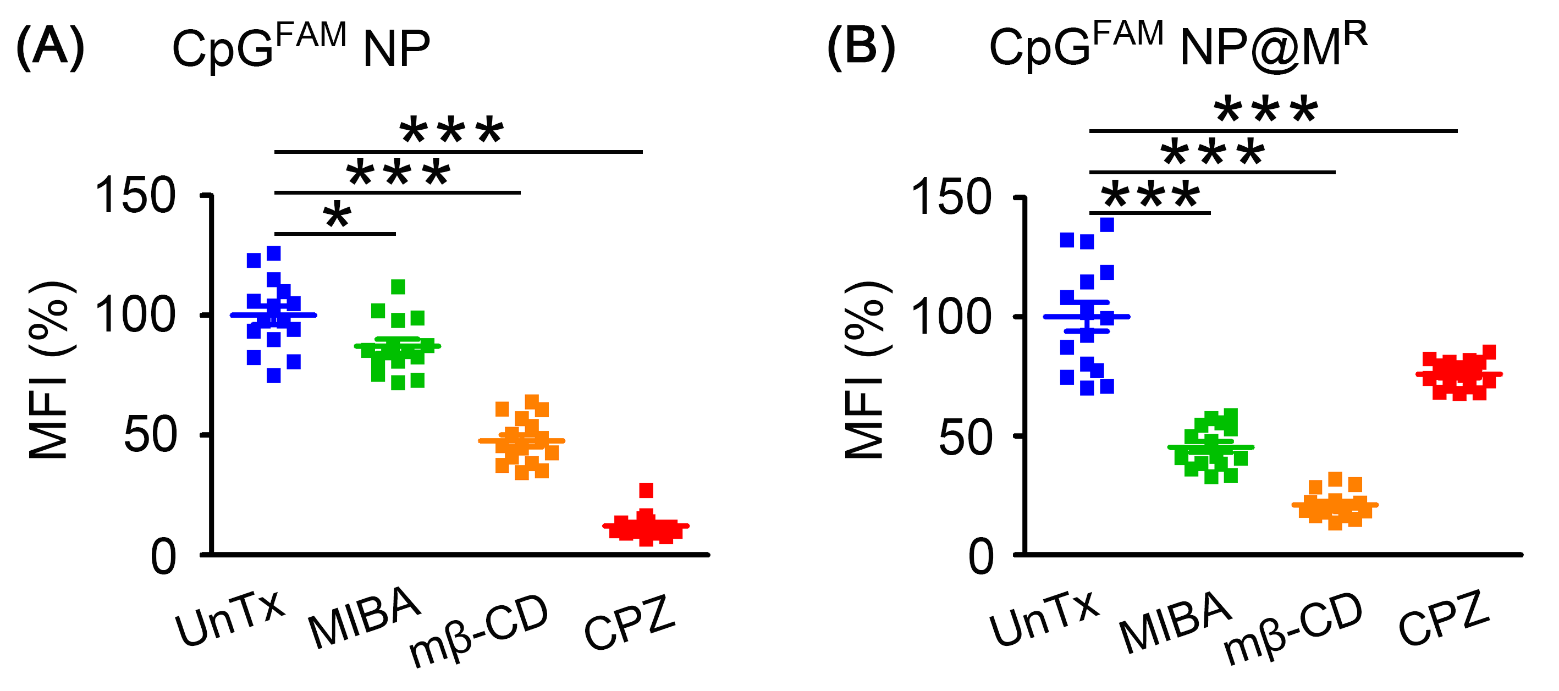


**Figure S5 Endocytic pathways of** **CpG NP and CpG NP@M^R^ in Raw264.7 cells.** Quantification of the cellular uptake efficiency of CpG^FAM^ NP (A) and CpG^FAM^ NP@M^R^ (B) in the presence of endocytosis inhibitors 5-(N-methyl-N-isobutyl) amiloride (MIBA), methyl-β-cyclodextrin (MβCD) or chlorpromazine hydrochloride (CPZ). Data are represented as mean ± s.e.m. (n = 15). Student’s *t*-test, **p* < 0.05, ****p* < 0.001.

**
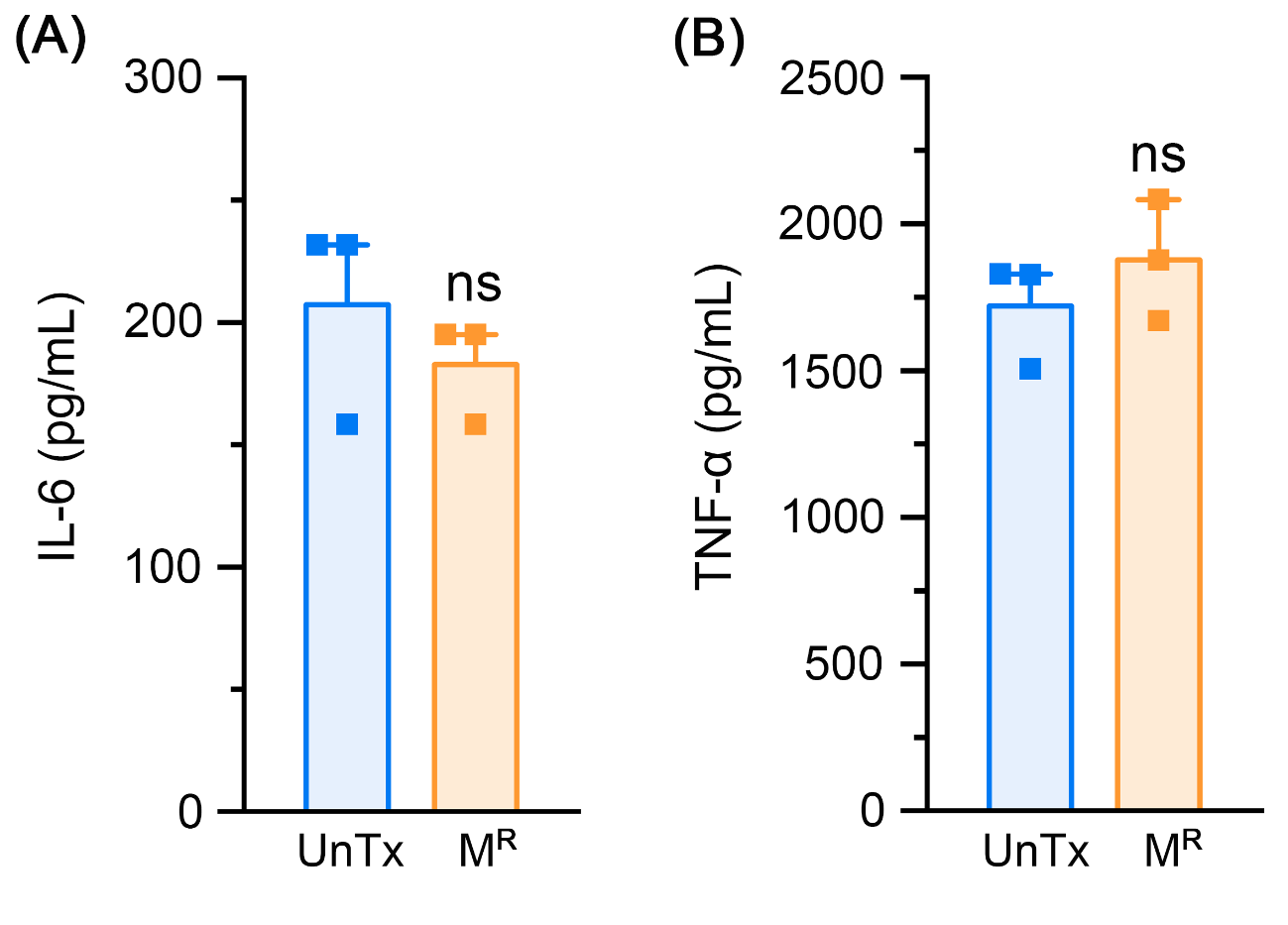
**

**Figure S6 The effects of cell membrane on cytokine secretion.** Secreted IL-6 (A) and TNF-α (B) from Raw264.7 cells in the absence or presence of the Raw264.7 cell membrane (M^R^) for 48 hours. The amount of M^R^ was equivlent to that bound to CpG NP. Data are represented as mean ± s.d. (n = 3). Student’s *t*-test, ns means not significant.


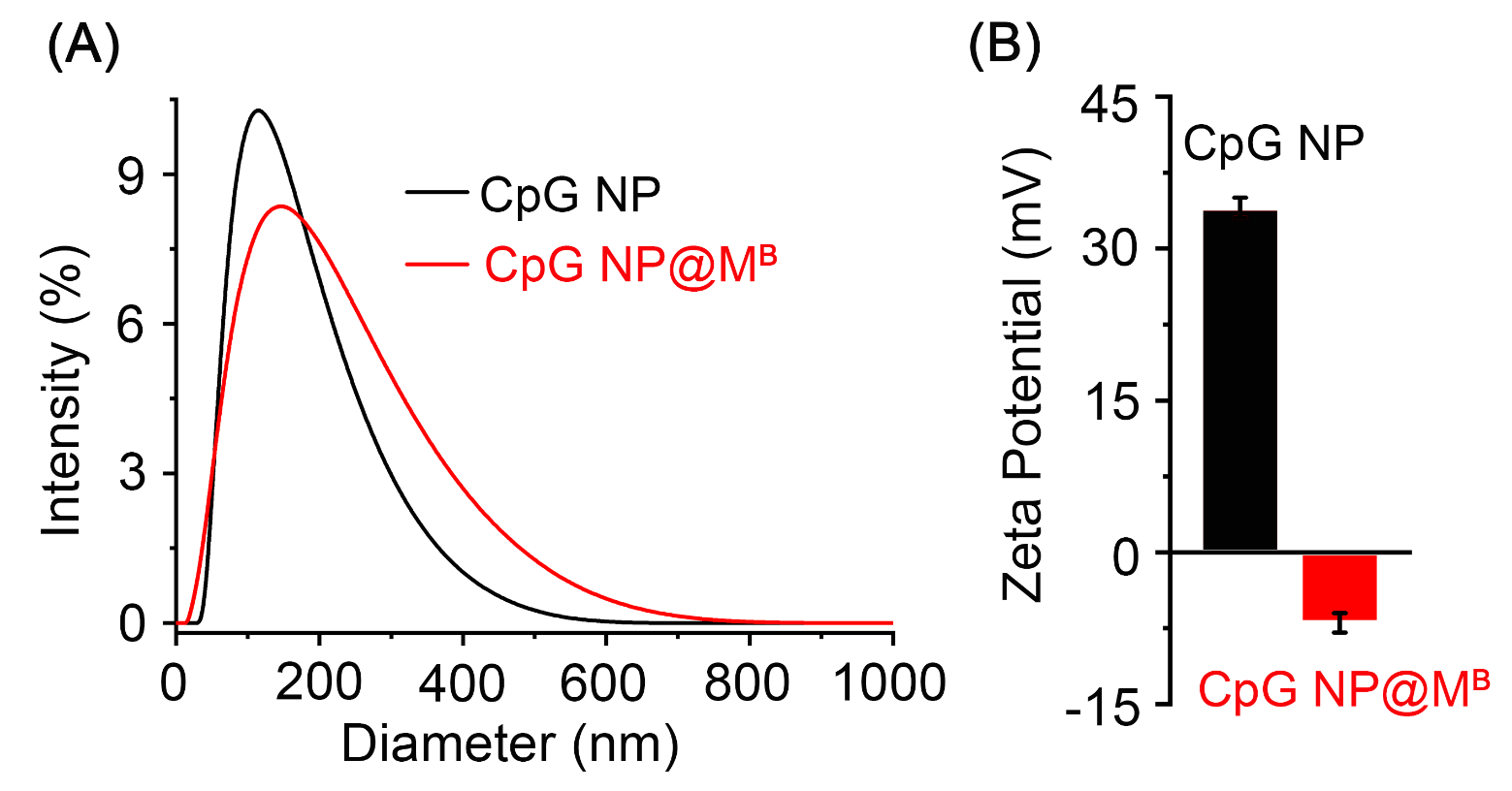


**Figure S7 Physicochemical properties of CpG NP and CpG NP@M^B^.** (A) DLS analysis for the hydrodynamic diameters of CpG NP and CpG NP@M^B^. (B) Zeta potetials of CpG NP and CpG NP@M^B^.


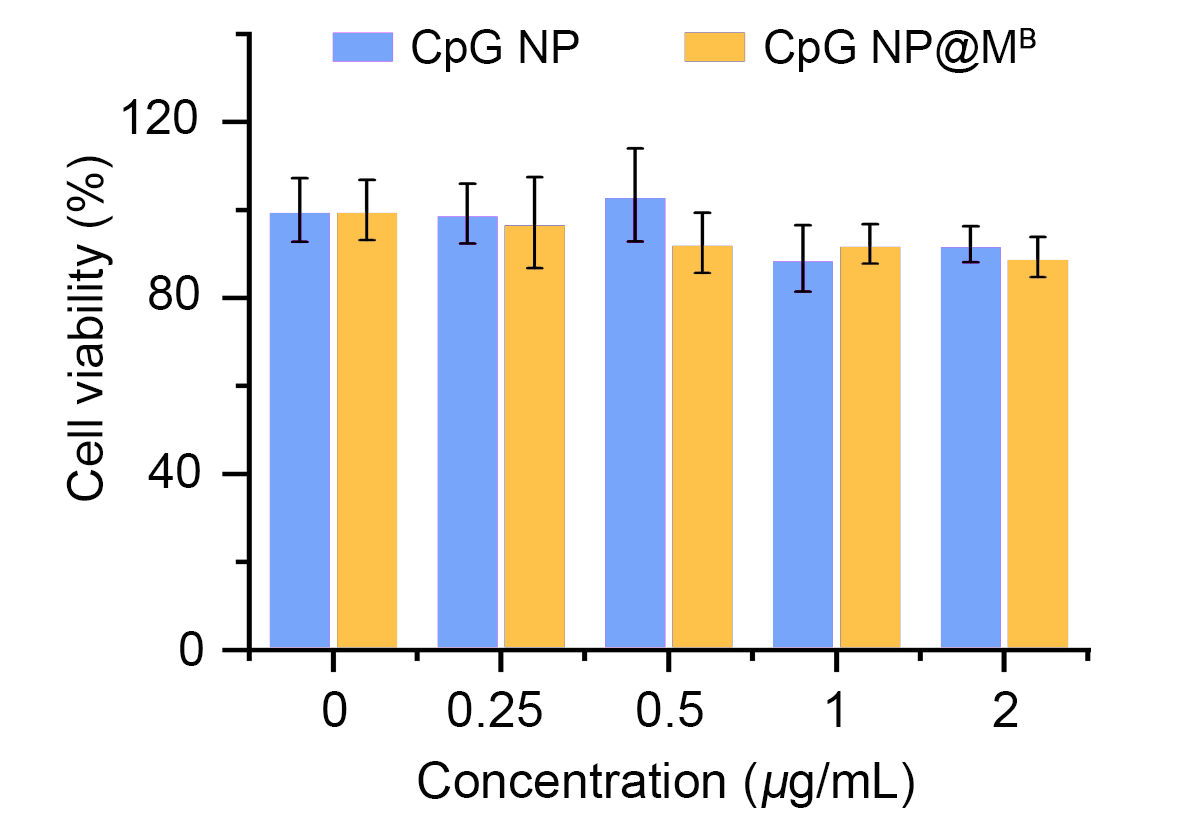


**Figure S8 Biocompatibility of CpG NP and CpG NP@M^B^ in BV-2 cells.** Cell viabilities of BV-2 cells incubated with CpG NP or CpG NP@M^B^ for 48 hours. Data are represented as mean ± s.d. (n=3).
